# *sodA* modulates *in vitro* and *in vivo* virulence of *Yersinia enterocolitica*

**DOI:** 10.3389/fmicb.2025.1643172

**Published:** 2025-09-15

**Authors:** Yingying Zhang, Yan Ma, Xiaodong Xia, Hao Li, Tong Jin, Xinru Meng, Xing Liu, Xuan Yang, Ruixue Xie, Li Li

**Affiliations:** ^1^College of Life Sciences and Medicine, Northwest University, Xi’an, China; ^2^College of Food Science and Engineering, Northwest A&F University, Yangling, Shaanxi, China; ^3^School of Biological and Food Processing Engineering, Huanghuai University, Henan, China; ^4^Department of Food Science and Nutrition, The Hong Kong Polytechnic University, Kowloon, Hong Kong SAR, China; ^5^Department of General Surgery, The Second Affiliated Hospital of Anhui Medical University, Hefei, China

**Keywords:** *Yersinia enterocolitica*, *sodA* gene, pathogenesis, reactive oxygen species, immune response

## Abstract

**Introduction:**

*Yersinia enterocolitica* is a significant foodborne Gram-negative pathogen causing gastroenteritis and systemic infections. Its virulence is attributed to its ability to withstand oxidative stress and evade host immunity. This study investigates the role of the sodA gene, which encodes manganese-dependent superoxide dismutase (Mn-SOD), in the pathogenicity of *Y. enterocolitica*.

**Methods:**

Using sodA knockout (Δ*sodA*) and complemented (Δ*sodA^C^*) strains, we assessed bacterial adhesion and invasion in Caco-2 epithelial cells, intracellular survival in RAW264.7 macrophages, and colonization in BALB/c mice. Inflammatory responses were evaluated through histopathology, immunohistochemistry (NF-κB p65), and qRT-PCR analysis of cytokine expression.

**Results:**

The Δ*sodA* mutant exhibited significantly reduced adhesion to and invasion of epithelial cells, impaired survival within macrophages, and decreased colonization in murine ileum and colon tissues. Furthermore, Δ*sodA* infection resulted in attenuated inflammatory responses, evidenced by lower expression of IL-1β, TNF-α, and NF-κB p65. Functional restoration was observed in the complemented strain, confirming the specific role of sodA.

**Discussion:**

These results demonstrate that sodA is essential for the full virulence of Y. enterocolitica, influencing oxidative stress resistance, host cell invasion, and modulation of immune responses. This study highlights sodA as a potential target for developing therapeutic strategies against *Y. enterocolitica* infections.

## 1 Introduction

*Yersinia enterocolitica* is widely distributed in various environmental settings and exhibits strong adaptability. Meat products are among the most frequent sources of contamination and serve as a primary vehicle for transmission (Zadernowska et al., 2014; Verbikova et al., 2018). *Y. enterocolitica* encounters moderate heating, refrigeration, pH variations, and osmotic stress during meat production, processing, storage, and transportation, which can lead to its entry into the host and subsequent inflammation. Compared to other bacteria, *Y. enterocolitica* shows greater resistance to environmental stressors such as cold stress and high osmotic pressure, which not only facilitates its survival in diverse environments but also enhances its pathogenic potential (Shoaib et al., 2019; Li et al., 2019). These adaptive mechanisms contribute to the bacterium’s ability to persist in food, water, and host tissues, ultimately influencing its transmission, colonization, and infection processes.

Yersiniosis caused by *Y. enterocolitica* is one of the most frequently reported zoonotic diseases in many European countries, following Campylobacteriosis and Salmonellosis in 2020 (European Food Safety Authority and European Centre for Disease Prevention and Control, 2021). It is primarily characterized by gastroenteritis but can also lead to conditions such as pseudo-renal disease, septicemia, reactive arthritis, and erythema nodosum (Bari et al., 2011; Özkütük, 2022). In the United States, the majority of yersiniosis cases are attributed to *Y. enterocolitica* infections, with approximately 90% of these cases linked to foodborne transmission (Petsios et al., 2016). The most common clinical manifestations of yersiniosis in humans include gastroenteritis and mesenteric lymphadenitis, though more severe conditions such as reactive arthritis, septicemia, and even death can occur (Adeolu et al., 2016; Rosner et al., 2013). *Y. enterocolitica* colonizes the terminal ileum and proximal colon by expressing the virulence plasmid pYV and secreting virulence factors that allow adhesion to intestinal epithelial cells (Fàbrega and Vila, 2012). In terms of function, *Y. enterocolitica* virulence genes can be roughly divided into three categories based on their association with adhesion, invasion, and effect on the host immune defense and disease processes (Wang et al., 2021). Like many other pathogens, *Y. enterocolitica* is susceptible to oxidative stress, which results from aerobic metabolism, environmental factors, and host immune responses that generate reactive oxygen species (ROS) (Liu et al., 2020). During oxidative bursts, ROS such as superoxide, hydrogen peroxide, and hydroxyl radicals are produced in large quantities. The excessive accumulation of these substances inhibits the growth and survival of the pathogen. To counteract the damaging effects of ROS, bacteria produce antioxidant compounds that neutralize endogenous and host-derived oxidative free radicals (Imlay, 2013).

Superoxide dismutase (SOD) is a widespread enzyme in various organisms that catalyzes the conversion of superoxide radicals into molecular oxygen or hydrogen peroxide. In order to successfully infect a host, *Y. enterocolitica* must overcome phagocytosis by neutrophils and macrophages. In this process, SOD plays a crucial role by reducing the bactericidal effects of ROS, thereby ensuring the survival and virulence of the pathogen within the host (Maurya and Namdeo, 2021). SOD enzymes are classified into different types based on the metal cofactors they require, including Mn-SOD, Fe-SOD, and Cu/Zn-SOD, which are encoded by the genes *sodA*, *sodB*, and *sodC*, respectively (Jair et al., 2019). Research has shown that inactivation of the *sodA* gene (encoding Mn-SOD) in bacteria is associated with reduced resistance to ROS (Turner et al., 2019). Additionally, numerous studies have demonstrated that the *sodA* gene is involved in regulating bacterial resistance to antimicrobial agents, acid tolerance, and biofilm formation (Smirnova et al., 2012; Bruno-Bárcena et al., 2010; Wang et al., 2018).

In this study, we investigated the impact of *sodA* gene deletion on the pathogenicity of *Y. enterocolitica* by assessing the adhesion and invasion ability of the mutant strain in intestinal epithelial cells and its survival and proliferation in macrophages. Using a BALB/c mouse model, we further examined bacterial colonization in organs and the gut, intestinal barrier integrity, and inflammation levels. These findings provide insights into the role of *sodA* in *Y. enterocolitica* infection.

## 2 Materials and methods

### 2.1 Strains and plasmids

The bacterial strains and plasmids used are listed in Table 1. *Y. enterocolitica* ATCC 23715 Amp (wild-type, WT) was cultivated in Luria-Bertani (LB) medium. *Escherichia coli* (*E. coli*) strains were cultured in LB medium. *Y. enterocolitica* strains were cultured at 26 °C. E. coli strains were cultured at 37 °C. The concentrations of antibiotics used in certain experiments were as follows: 20 μg/mL of chloramphenicol (Cm) and 100 μg/mL of ampicillin (Amp) in the experiment.

**Table 1:** Bacterial strains and plasmids used in the study.

Strain or plasmid	Relevant characteristics	Reference or source
Strains		
*Y. enterocolitica*		
ATCC 23715/Amp	WT, Ampcillin-induced mutant of *Y. enterocolitica* ATCC 23715, Amp^r^	Laboratory collection
*ΔsodA*	*sodA* gene deletion of ATCC 23715, Amp^r^	This study
*ΔsodA^C^*	*ΔsodA* strain complemented with pACYC184-*sodA*, Amp ^r^, Cm^r^	This study
*E. coli*		
S17–1 (λpir)	Tpr Smr recA thi pro rK-mK-RP4:2-Tc: MuKm Tn7 λ pir	Xu et al. (2018)
DH5*α*	F-supE44 ΔlacU169 hsdR17 recA1 endA1 gyrA96 thi- 1 relA1	Purchased from Accurate Biology (AG, Hunan, China)
Plasmids		
pDM4	Suicide vector, *pir* dependent, R6K, *sacBR*, Cm^r^	Dai et al. (2018)
pDM4-*sodA*	Plasmid for deletion of *sodA* gene, Cm^r^	This study
pACYC184	Cloning vector, p15A ori, Cm^r^, Tc^r^	Ma et al. (2021)
pACYC184-*sodA*	*sodA* gene with its promoter region clones into pACYC184, Cm^r^	This study

### 2.2 Construction of *sodA* deletion mutant and complemented strain

SacB-based allelic exchange was used to create the in-frame deletion mutant as previously described (Ma et al., 2021). The primers *sodA*-UP-F/*sodA*-UP-R and *sodA*-DOWN-F/*sodA*-DOWN-R were used to amplify the upstream and downstream regions around the *sodA* gene (Table 1). The primers *sodA*-UP-F/*sodA*-DOWN-R were used to splice the PCR products using overlap extension-PCR. Linearized pDM4 plasmid and the purified *sodA*-UD fragment were ligated using the Gibson assembly method. After preparing the reaction system according to the protocol, the mixture was incubated at 50 °C for 1 h to facilitate ligation and fusion. The resulting fusion product was cloned into pDM4 and digested with Xho I to obtain the recombinant expression vector pDM4-*sodA*. Following purification, the plasmid was transformed into *E. coli* S17-1 (λpir) by heat shock and transferred into the WT. Single crossover recombination occurred on LB agar plates supplemented with Cm, and double crossover recombination events occurred on LB agar plates supplemented with 10% sucrose. Ultimately, the Δ*sodA* mutant was verified using the primers *sodA*-IN-F/ *sodA*-IN-R and *sodA*-UP-F/*sodA*-DOWN-R. The primers *sodA*-Hind III-F and *sodA*-BamH I-R were used to amplify the *sodA* gene and its promoter region by PCR (Supplementary Table S1). The PCR product was inserted into Hind III and BamH I double-digested pACYC184, resulting in the complemented plasmid pACYC184-*sodA*. This plasmid was transferred into Δ*sodA*, and the complemented isolates were selected on LB agar plate containing Cm and Amp. Eventually, the complemented strain Δ*sodA^C^* was confirmed using the primers *sodA*-IN-F and *sodA*-IN-R (Supplementary Figure S1).

### 2.3 Phenotypic profiling of *sodA* mutant

The effect of *sodA* deletion on acid tolerance and oxidative stress resistance were evaluated based on the method of Ma et al. (2021) with minor modifications. Overnight bacterial cultures of WT, Δ*sodA*, and Δ*sodA^C^* strains were diluted to 1 × 10^7^ CFU/mL using LB broth. 300 μL bacterial suspension was inoculated into 30 mL LB broth (pH 4.0) and incubated at 26 °C for 1 h. Serial dilutions were plated on LB agar, with viable counts enumerated after 48 h incubation at 26 °C. Cell suspensions were adjusted to OD_600_ = 0.5 in LB broth. Aliquots (100 μL) were lawn-cultured on LB agar. Sterile Oxford cups were placed on agar, filled with 200 μL 10% (v/v) H_2_O_2_, and incubated 24 h at 26 °C. Inhibition zone diameters were measured with vernier calipers. Bacterial suspensions (OD_600_ = 0.5) were diluted 1:100 in LB broth containing 5 mM H_2_O_2_. After 1 h incubation at 26 °C, cells were serially diluted in PBS and viable counts determined by spread plating.

### 2.4 Biofilm metabolic activity quantification and morphological characterization

This study aimed to comprehensively assess *sodA*-dependent regulation of biofilm formation through quantitative biomass, metabolic activity, and architectural analyses as previously described (Bai et al., 2019). Overnight cultures of WT, Δ*sodA*, and Δ*sodA^C^* strains were adjusted to OD_600_ = 0.5. Aliquots (200 μL) were dispensed into 96-well polystyrene plates (triplicate wells per strain), with sterile LB as blank control. After 48 h static incubation at 26 °C, planktonic growth was measured at OD_630_. Biofilm biomass was quantified via crystal violet staining (OD_570_) to calculate Specific Biofilm Formation (SBF) index using the formula: SBF = (OD_570 nm_−OD_control_)/(OD_630 nm_−OD_control_). Biofilms grown as above were incubated with 0.5 mg/mL MTT (200 μL/well) for 4 h at 26 °C. Formazan crystals were dissolved in DMSO (150 μL/well) with gentle agitation. Metabolic activity was measured at OD_490_. Biofilms were formed on glass coverslips (Ø10 mm) in 24-well plates containing 2 mL bacterial suspension (OD_600_ = 0.5). After 48 h at 26 °C, coverslips were crystal violet-stained (0.4%, 20 min), washed, air-dried, and examined at 400 × magnification. Then fixed in 2.5% glutaraldehyde (4 °C overnight), dehydrated in ethanol gradient (30–100%), critical-point dried, gold-sputtered, and imaged by FESEM at 4,000×.

### 2.5 Adhesion and invasion assay of epithelial cells

Caco-2 cells were cultured in DMEM supplemented with 10% FBS, 1% non-essential amino acids, and 1% antibiotic-antimycotic solution at 37 °C with 5% CO₂. Once 85% confluent, cells were washed with PBS, digested with 0.25% trypsin–EDTA, and passaged at a 1:5 ratio into new flasks. The adhesion and invasion assays for Caco-2 cells were performed with reference to the method of Medeiros et al. (2021) with minor modifications. Briefly, Caco-2 cells were digested and diluted to 1 × 10^5^ cells/mL, then seeded into 24-well plates and incubated overnight. Overnight bacterial cultures of WT, Δ*sodA*, and Δ*sodA^C^* strains were diluted to 1 × 10^7^ CFU/mL using DMEM. After gently washing the Caco-2 cells, 1 mL of bacterial suspension was added to each well, followed by centrifugation (600 × g, 5 min). Samples were incubated at 37 °C with 5% CO_2_ for 2 h, after which the supernatant was discarded, and the cells were washed with sterile PBS.

For the adhesion assay, 0.1% (v/v) Triton X-100 was added to each well to lyse the cells at 4 °C for 20 min. The bacterial suspension was pipetted to homogenize, serially diluted, and plated. Colony counts were recorded after incubation at 26 °C for 48 h. For the invasion assay, 1 mL of medium containing gentamicin (100 μg/mL) was added to each well and incubated at 37 °C with 5% CO_2_ for 45 min. After incubation, the cells were washed, lysed, and plated to quantify the number of invasive bacteria.

### 2.6 Survival and proliferation in macrophages

The survival and proliferation abilities of *Y. enterocolitica* in RAW264.7 macrophages after *sodA* gene deletion were evaluated based on the method of Jin et al. (2024) with minor modifications. The RAW264.7 cell culture process was similar to that described in Section 2.3, except the digestion time was 3.5 min. After digestion, complete medium was added to halt digestion, and the cell suspension was passaged at a ratio of 1:5 into new flasks. RAW264.7 cells were cultured similarly to Caco-2 cells. RAW264.7 cells were seeded in 24-well plates at 1 × 10^5^ cells/mL. Overnight bacterial cultures of WT, Δ*sodA*, and Δ*sodA^C^* strains (~1 × 10^7^ CFU/mL) were added to each well, centrifuged (600 × g, 5 min), and incubated at 37 °C with 5% CO_2_ for 45 min. The supernatant was then discarded, and the cells were washed once with PBS.

For the survival assay, 1 mL of medium containing gentamicin (100 μg/mL) was added to each well, and the samples were incubated at 37 °C with 5% CO_2_ for 30 min. After incubation, the cells were lysed using 0.1% Triton X-100, and bacterial colonies were quantified by plating. For the proliferation assay, 1 mL of medium containing gentamicin (10 μg/mL) was added to each well and incubated for 24, 48, and 72 h. Samples were collected every 24 h, washed, lysed, and plated to quantify bacterial proliferation.

### 2.7 Mice model and experimental procedures

Sixty specific pathogen-free (SPF) female BALB/c mice (6 weeks old) were used in this study. The animal experiments in this study were conducted in strict accordance with the Guide for the Care and Use of Laboratory Animals (8th edition, ISBN-10: 0-309-15396-4) and the Laboratory Animal Management Regulations of Northwest A&F University. The mice were housed in a controlled environment at 25 °C with free access to food and water. The housing conditions were kept dry and clean, with bedding changed every 2 days, and five mice were housed per cage.

The mice were randomly divided into four groups: control (Control), wild-type strain infection (WT), *sodA* deletion strain infection (Δ*sodA*), and *sodA* complemented strain infection (Δ*sodA^C^*). Based on preliminary experiments, the minimum infectious dose of *Y. enterocolitica* ATCC 23715 causing clinical symptoms in BALB/c mice was determined to be approximately 1 × 10^9^ CFU. For this study, the infection dose was set at 4 × 10^9^ CFU. WT, Δ*sodA*, and Δ*sodA^C^* bacterial suspensions were prepared as described in Section 2.1, and the concentration was adjusted to 4 × 10^10^ CFU/mL. After a 7-day acclimation period, the mice were orally gavaged with 100 μL of the bacterial suspensions (WT, Δ*sodA*, or Δ*sodA^C^*). The control group received 100 μL of sterile PBS. Mice were weighed daily after infection. On day 5 post-infection, mice were anesthetized with 3.5% chloral hydrate via intraperitoneal injection, euthanized by cervical dislocation, and dissected using sterile instruments. Liver, spleen, kidneys, and intestinal tissues (from duodenum to rectum) were collected. The tissues were gently washed with pre-cooled sterile saline and fixed in 4% paraformaldehyde for at least 24 h. Remaining tissues were snap-frozen in liquid nitrogen and stored at −80 °C for further analysis.

### 2.8 Assessment of *Yersinia enterocolitica* colonization post-infection

Fecal samples were collected at 0, 1, 2, 3, 4 and 5 days post-infection, weighed, and subjected to tenfold serial dilution in PBS. A 100 μL aliquot of each dilution was plated on CIN-1 agar and incubated at 26 °C for 24 h. On day 5 post-infection, mice were euthanized as described above. The kidneys, liver, spleen, ileum, and colon were harvested, washed with pre-cooled PBS, and blotted dry with filter paper. Samples were weighed and placed into pre-cooled microcentrifuge tubes. Tissue homogenates were prepared using a tissue homogenizer with PBS based on the weight of the sample. Homogenates were serially diluted in PBS, and 100 μL of each dilution was plated on CIN-1 agar and incubated at 26 °C for 24 h. The number of characteristic colonies on CIN-1 agar was recorded and subjected to statistical analysis.

### 2.9 Histopathology

The tissues for histological examinations were fixed overnight with 4% paraformaldehyde overnight and paraffin- embedded. After sliced into 3 μm sections, the tissue sections were then dewaxed and stained with hematoxylin and eosin (H&E; Solarbio) and analyzed by a light microscope (Leica, Wetzlar, Germany).

### 2.10 Immunohistochemical staining

The level of NF-κB p65 in the ileum was assessed using immunohistochemical staining (Jin et al., 2022). Briefly, paraffin sections were treated with dewaxing, antigen retrieval, and blocking endogenous peroxidase, followed by a 30 min incubation with 10% rabbit serum for blocking. The sections were incubated with the primary antibody at 4 °C overnight followed by a 50 min incubation with the secondary antibody, and they were finally stained with DAB and counterstained with hematoxylin. The results were observed by fluorescence microscopy (Leica, Wetzlar, Germany), and the mean optical density was evaluated by Image J (Version 1.8.0.112).

### 2.11 qRT-PCR

Total RNA was extracted by a Steady Pure RNA extraction kit (AG, Changsha, China), followed by reverse transcription into cDNA by an Evo M-MLV reverse transcription kit. The qRT-PCR analysis was conducted using an IQ5 system (Bio-Rad). The primer sequence, along with GAPDH as the reference gene, is presented in Supplementary Table S2.

### 2.12 Statistical analysis

The data are presented as means ± SD. SPSS 19.0 was used for one-way analysis of variance. The data were analyzed using one-way ANOVA. *: *p* < 0.05; **: *p* < 0.01. #: *p* < 0.05; ##: *p* < 0.01; §: *p* < 0.05; §§: *p* < 0.01. Primer design was performed using Primer 5.0 software.

## 3 Results

### 3.1 Construction and validation of *sodA* knockout and complementation strains

Genomic DNA from *Y. enterocolitica* ATCC 23715 was used to amplify the upstream and downstream homologous arms of the *sodA* gene, yielding 947 bp and 945 bp fragments, respectively, confirmed by agarose gel electrophoresis. The fused homologous arm fragment (1892 bp) was successfully assembled into the XhoI-digested pDM4 vector using Gibson assembly and transformed into *E. coli* S17-1 (λpir). Positive clones were identified by PCR, confirming the construction of the recombinant plasmid pDM4-*sodA*. To verify single colonies on sucrose plates, *sodA*-UP-F/*sodA*-DOWN-R primers and *sodA*-IN-F/R primers were utilized. The WT U + *sodA*+D fragment measured 2,407 bp, while the deletion strain U + D fragment was 1892 bp. PCR amplification was detected for the deletion strain, confirming the successful knockout of the *sodA* gene using the sucrose-sensitive suicide vector pDM4. The *sodA* gene fragment, including its promoter, was subsequently cloned into the HindIII and BamHI-digested pACYC184 vector through double digestion. PCR amplification with *sodA*-HindIII-F/*sodA*-BamHI-R primers yielded a 2,409 bp fragment. The positive recombinant strain successfully amplified a 1734 bp band, consistent with the expected result, indicating successful construction of the recombinant plasmid pACYC184-*sodA*. Complementation was verified by PCR screening using *sodA*-IN-F/R primers. Both the WT and complementation strains produced a 601 bp *sodA* fragment, confirming the successful construction of the *sodA* complementation strain.

### 3.2 Oxidative stress susceptibility of *sodA* mutant

**Figure 1:**
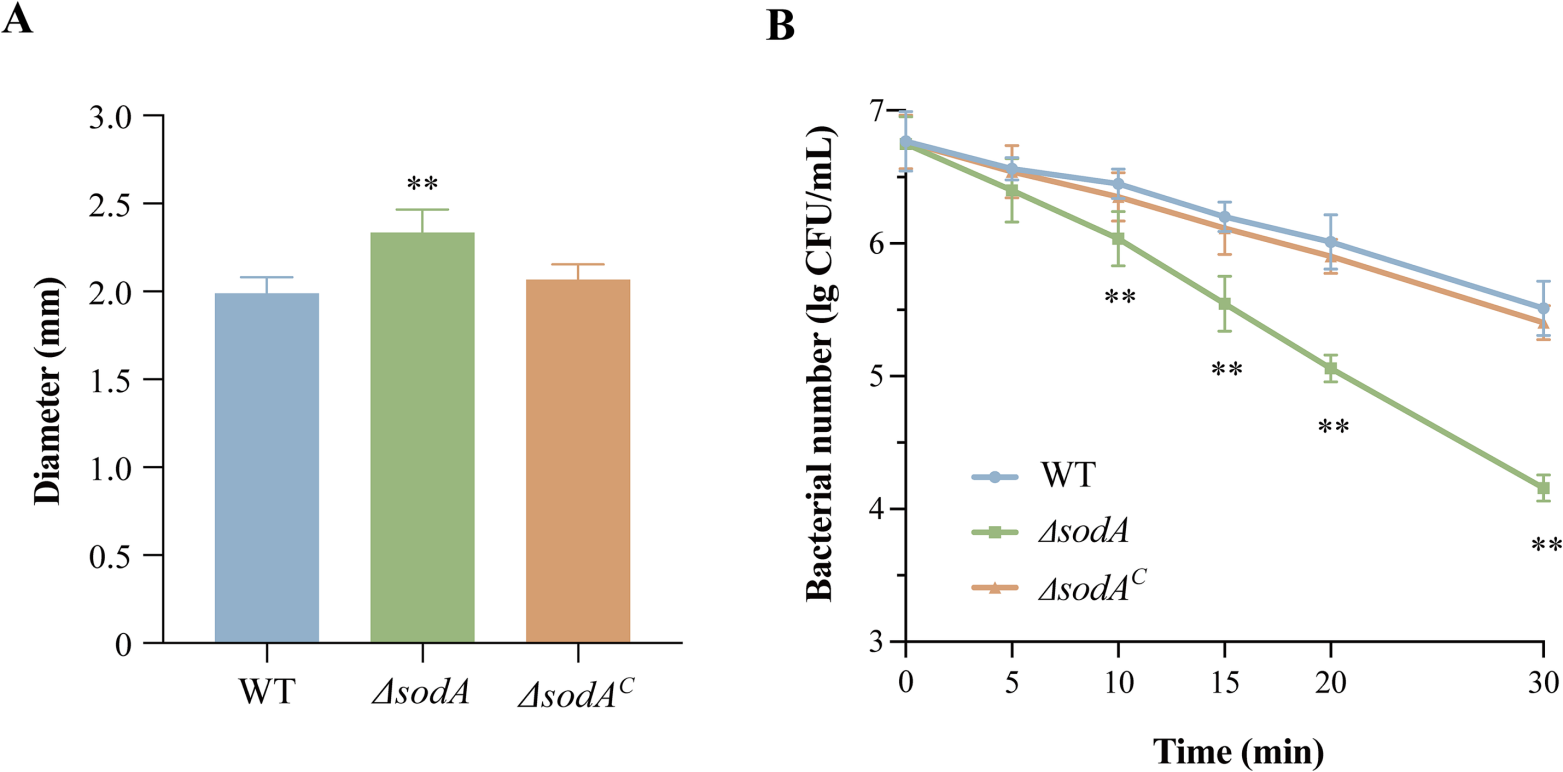
Deletion of *sodA* compromises oxidative stress resistance in *Y. enterocolitica*. **(A)** H₂O₂ sensitivity assessed by agar well diffusion assay. **(B)** Time-dependent survival under 5 mM H₂O₂. * Indicates a comparison with the WT group (*: *p* < 0.05; **: *p* < 0.01). *n* = 3 biological replicates.

In agar well diffusion assays (Figure 1A), Δ*sodA* displayed a 23.3% larger inhibition zone (2.33 ± 0.07 mm vs. WT: 1.89 ± 0.05 mm; *p* < 0.01) after 24 h incubation at 26 °C, indicating heightened H_2_O_2_ sensitivity. The complemented strain (Δ*sodA^C^*) showed no significant difference from WT (*p* > 0.05). As shown in Figure 1B, all strains exhibited significantly reduced viability under 5 mM H₂O₂ exposure. While Δ*sodA* showed comparable survival to WT and complemented Δ*sodA^C^* at 5 min (*p* > 0.05), it demonstrated progressively diminished viability at extended timepoints (10, 15, 20, and 30 min), with statistically significant reductions versus controls (*p* < 0.05). These data establish that *sodA* deletion compromises oxidative stress tolerance in *Y. enterocolitica*, confirming its essential role in ROS detoxification.

### 3.3 *sodA*-dependent biofilm development and metabolic regulation

**Figure 2:**
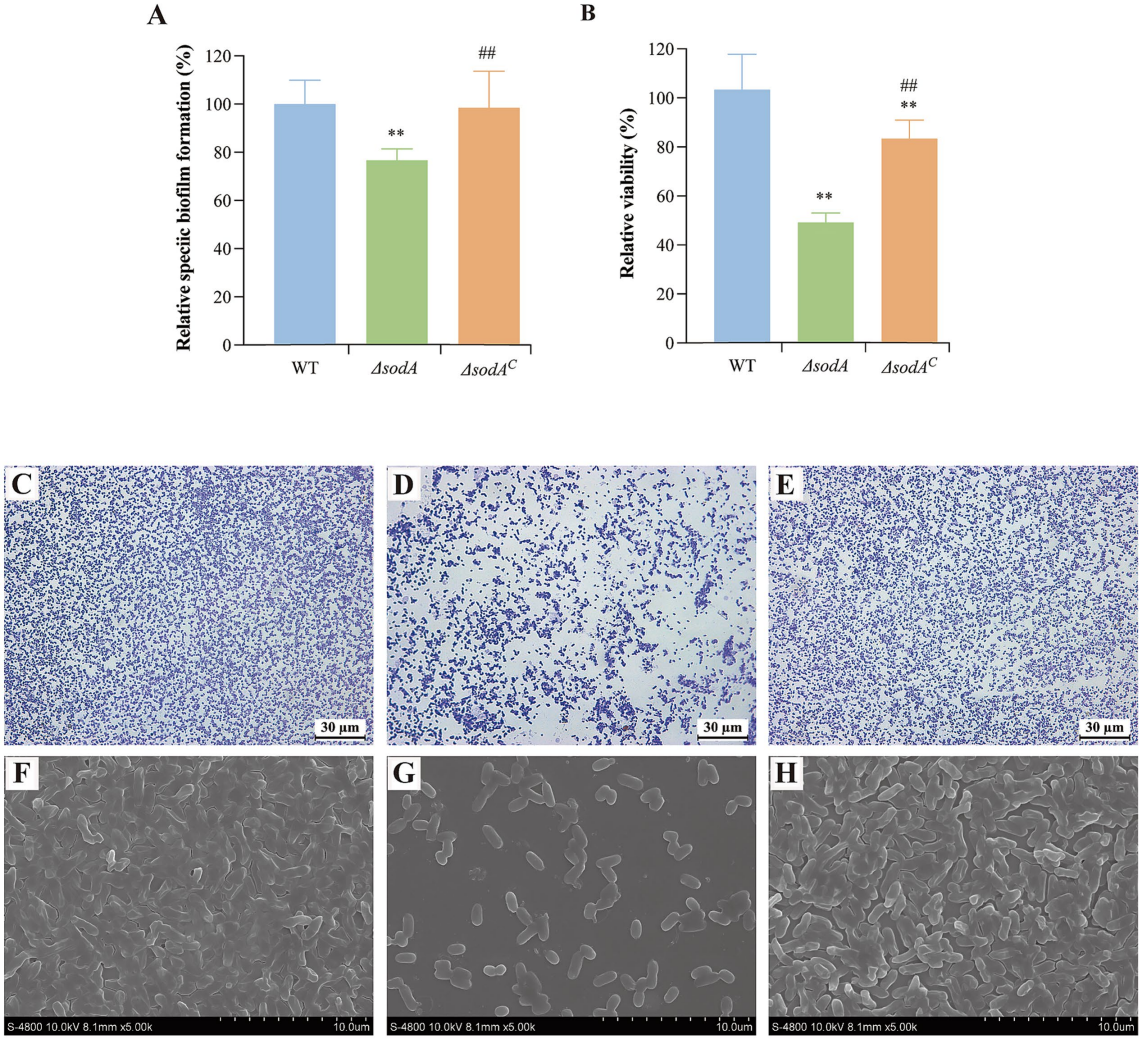
*sodA* deletion impairs biofilm biomass formation and metabolic activity in *Y. enterocolitica*. **(A)** Quantitative biofilm biomass measured by crystal violet staining. **(B)** Biofilm metabolic activity assessed by MTT assay. * Indicates a comparison with the WT group (*: *p* < 0.05; **: *p* < 0.01); # indicates a comparison with the Δ*sodA* group (#: *p* < 0.05; ##: *p* < 0.01). *n* = 3 biological replicates. Optical microscope images of WT **(C)**, Δ*sodA* **(D)** and Δ*sodA^C^* **(E)** biofilms (400 × magnification). Scanning electronic images of WT **(F)**, Δ*sodA* **(G)** and Δ*sodA^C^* **(H)** bilfilms (4,000 × magnification). All images are representative of ≥3 biological replicates.

Figure 2A demonstrates that *sodA* deletion significantly impaired biofilm biomass formation (*p* < 0.01). The Δ*sodA* mutant exhibited 29.37% reduction in crystal violet-stained biomass, while Δ*sodA^C^* partially restored this phenotype. MTT metabolic assays (Figure 2B) revealed severely compromised biofilm viability in Δ*sodA* (48.09% reduction vs. WT; *p* < 0.01). Δ*sodA^C^* showed significantly higher metabolic activity than Δ*sodA* (*p* < 0.01), indicating *sodA*-mediated regulation of biofilm metabolic processes. Morphological analyses corroborated the quantitative biofilm defects: wild-type (WT) biofilms developed dense, stratified architectures with robust surface adherence (Figures 2C,F), whereas the Δ*sodA* mutant formed sparse monolayers exhibiting compromised structural cohesion (Figures 2D,G). Δ*sodA^C^* restored the biofilm morphology, yielding consolidated multilayered structures (Figures 2E,H). This visual evidence from both light and field-emission scanning electron microscopy consistently demonstrates *sodA* essential role in maintaining biofilm architectural integrity.

### 3.4 Impact of *sodA* gene deletion on adhesion to and invasion of host cells

**Figure 3:**
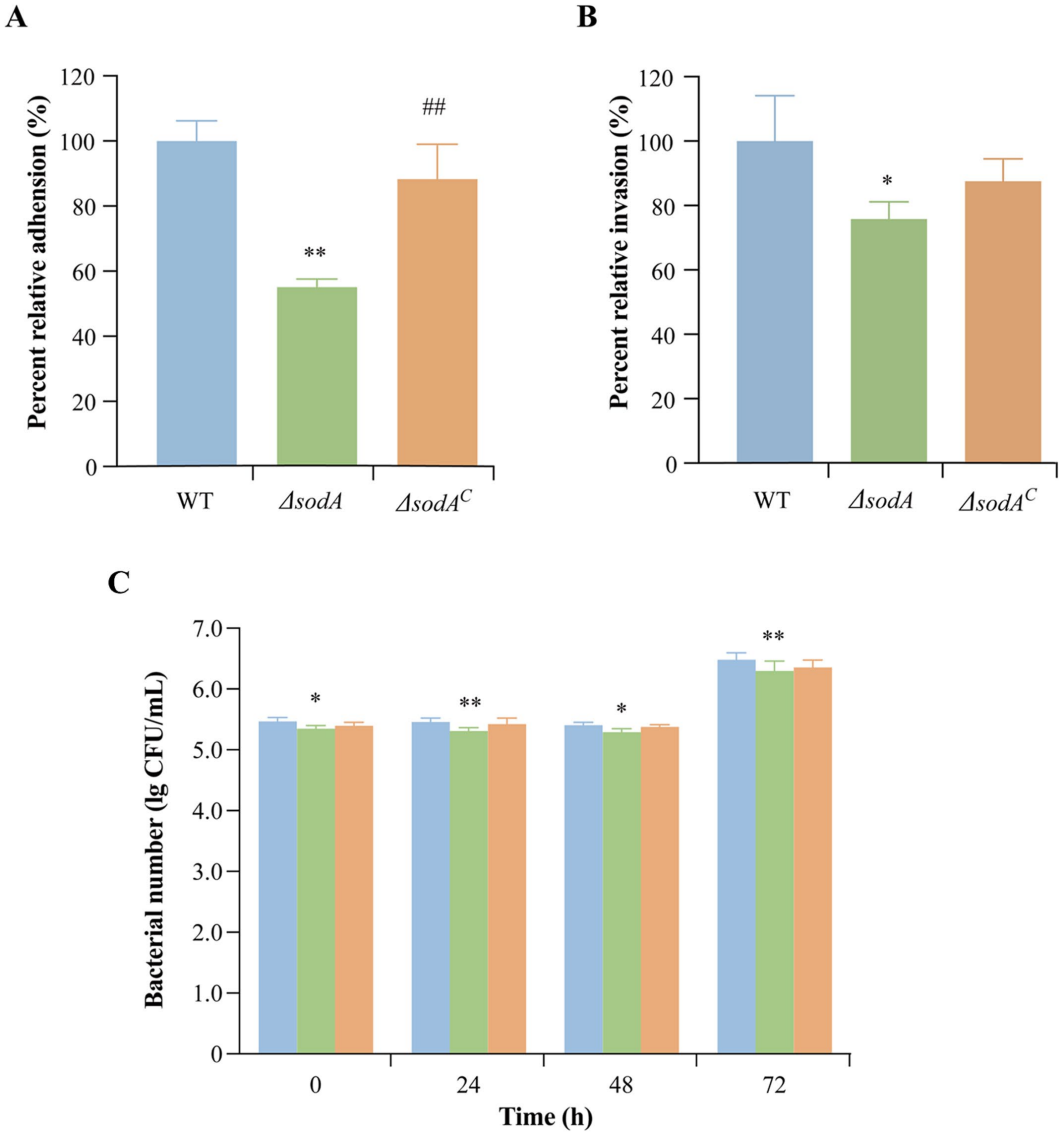
Effect of *sodA* gene deletion on adhesion **(A)** and invasion **(B)** of Caco-2 cells by *Y. enterocolitica*. **(C)** Effect of *sodA* gene deletion on survival and replication of RAW264.7 macrophage cells by *Y. enterocolitica*. * Indicates a comparison with the control group (*: *p* < 0.05; **: *p* < 0.01); # indicates a comparison with the WT group (#: *p* < 0.05; ##: *p* < 0.01). *n* = 3 biological replicates.

*Y. enterocolitica* initiates infection through adhesion to and invasion of host cells. As shown in Figure 3A, the adhesion rates of the Δ*sodA* and Δ*sodA^C^* strains to Caco-2 cells were reduced to 55.14 and 88.29%, respectively, compared to the WT strain. Upon complementation of the *sodA* gene, the adhesion ability of *Y. enterocolitica* was significantly restored (*p* < 0.01). Additionally, *sodA* deletion suppressed the bacterial invasion of Caco-2 cells, with invasion rates decreasing to 75.84 and 87.57% (Figure 3B). These results indicate that the *sodA* gene plays a critical role in the adhesion and invasion of host cells by *Y. enterocolitica*.

### 3.5 Survival and proliferation in macrophages

RAW264.7 cells, a mouse monocyte–macrophage leukemia cell line derived from BALB/c mice, were used to evaluate intracellular survival. As shown in Figure 3C, the Δ*sodA* strain exhibited higher phagocytosis sensitivity in RAW264.7 macrophages compared to the WT and Δ*sodA^C^* strains.

### 3.6 Weight changes induced by *Yersinia enterocolitica* infection

**Figure 4:**
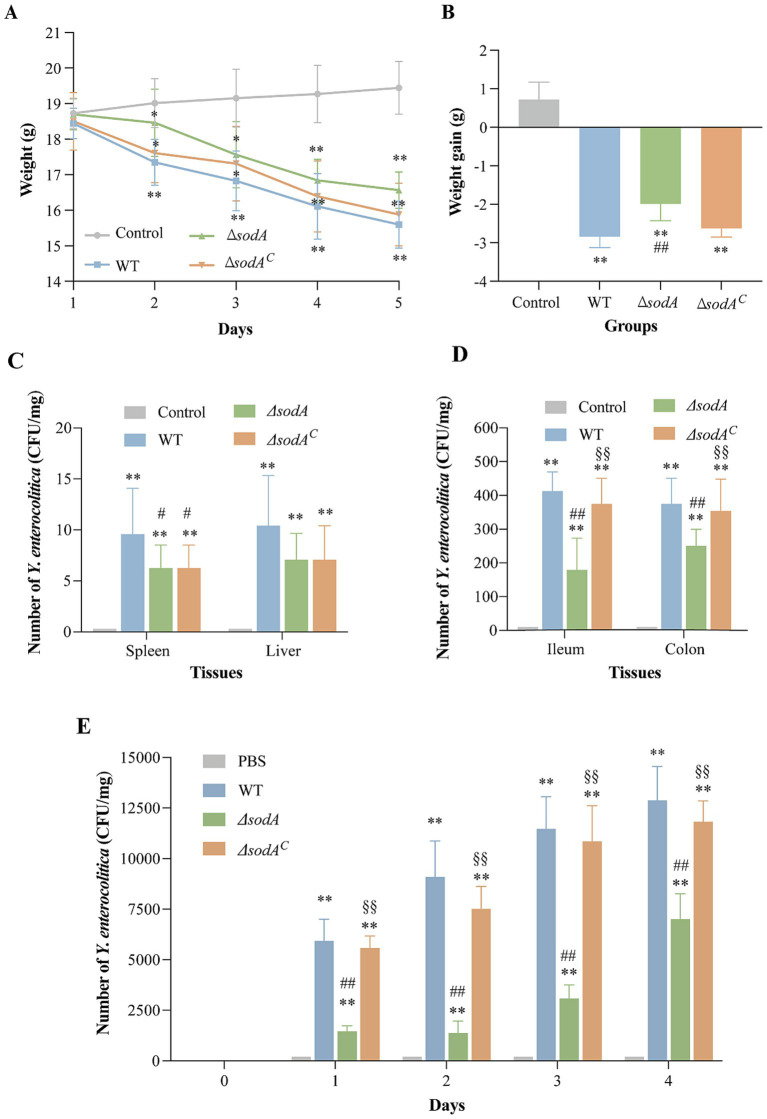
Weight changes 5 days after infection with *Y. enterocolitica* **(A)** and weight increment of mice **(B)**. Effect of *sodA* deletion on colonization ability of *Y. enterocolitica* in mice. Number of *Y. enterocolitica* in spleen and liver **(C)**, ileum and colon **(D)**, fecal in 4 days after infection **(E)**. * Indicates a comparison with the control group (*: *p* < 0.05; **: *p* < 0.01); # indicates a comparison with the WT group (#: *p* < 0.05; ##: *p* < 0.01); § indicates a comparison with the Δ*sodA* group (§: *p* < 0.05; §§: *p* < 0.01). *n* = 15 biological replicates in weight change and increment of mice. *n* = 6 biological replicates in bacterial colonization counting assay.

Figures 4A,B illustrate body weight changes in mice over 5 days of *Y. enterocolitica* infection. Control mice administered sterile PBS showed continuous weight gain, whereas significant weight loss was observed in the WT-infected group on day 2 (*p* < 0.01). Similarly, mice infected with Δ*sodA* and Δ*sodA^C^* strains also showed notable weight reduction (*p* < 0.05). Over the five-day period, weight loss was −2.84 ± 0.29 g, −1.99 ± 0.43 g, and −2.63 ± 0.22 g in the WT, Δ*sodA*, and Δ*sodA^C^* groups, respectively, with significantly greater weight loss in the WT group compared to the Δ*sodA* group (*p* < 0.01).

### 3.7 Deletion of *sodA* gene reduced the bacterial loads of *Yersinia enterocolitica* in mice

Using CIN-1 selective media, *Y. enterocolitica* colonies were quantified in various tissues of mice (Figures 4C,D). No colonies were detected in the tissues of control mice. Infected groups showed low bacterial colonization in the liver and spleen. The WT and Δ*sodA^C^* groups exhibited significantly higher colonization in ileum and colon tissues (~400 CFU/mg, *p* < 0.01) compared to Δ*sodA* (179 CFU/mg and 258 CFU/mg, respectively). Fecal bacterial counts after 5 days of infection (Figure 4E) were significantly lower in the Δ*sodA* group compared to the WT and Δ*sodA^C^* groups (*p* < 0.01). These findings suggest that *sodA* gene deletion reduces the number of *Y. enterocolitica* colonization in the mouse gut.

### 3.8 *sodA* affected the ability of pathogen to induce histopathological damage

**Figure 5:**
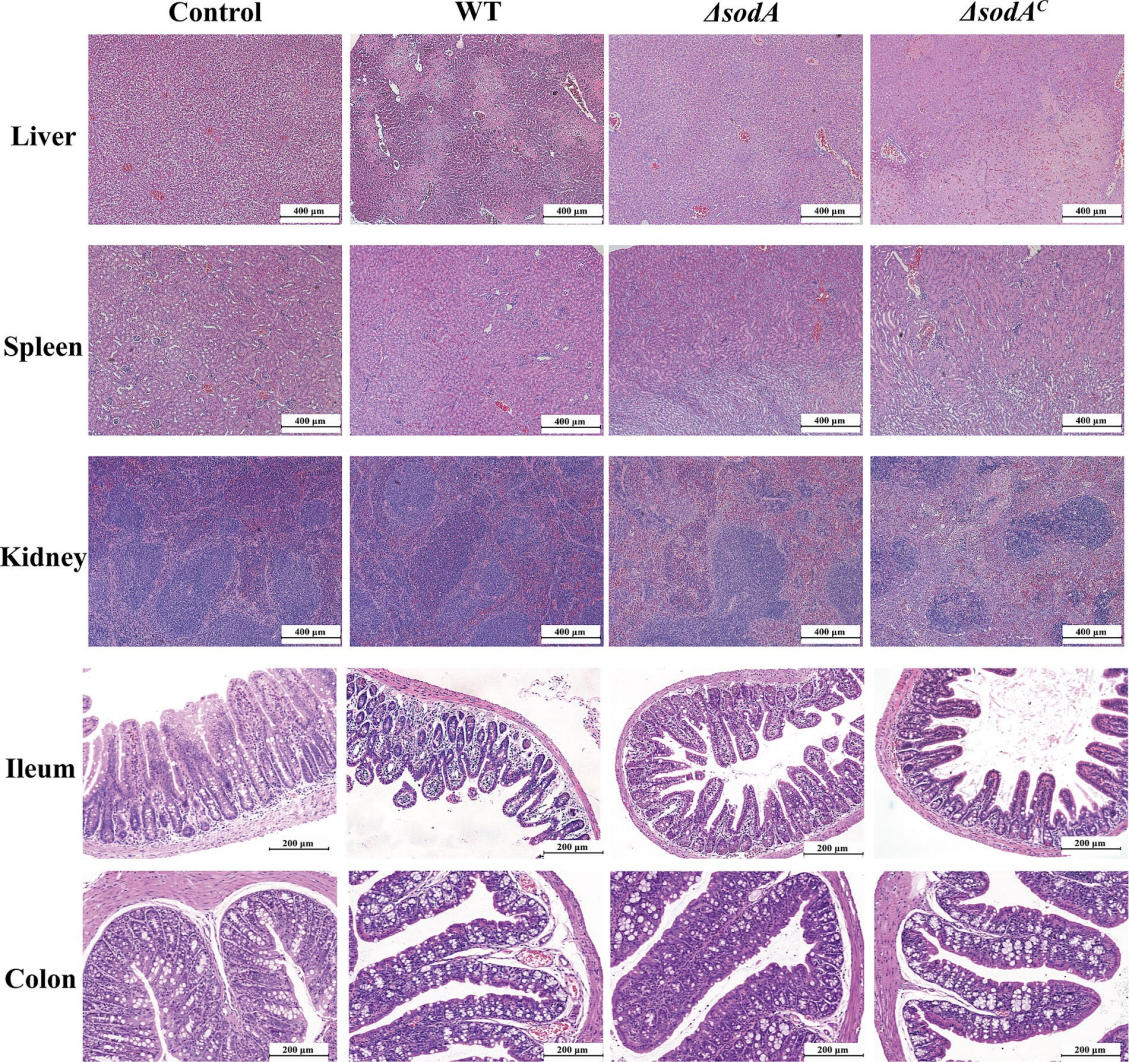
H&E staining of liver, spleen, kidney (100×), ileum and colon tissue of mice (200×). *n* = 3 biological replicates.

H&E staining results of mouse tissues are shown in Figure 5. Control group liver tissue displayed normal cell morphology, while the WT group exhibited severe shrinkage and nuclear chromatin condensation. Mice infected with Δ*sodA* strains showed slight shrinkage with otherwise normal morphology, while those infected with Δ*sodA^C^* strains displayed moderate shrinkage. Similar trends were observed in kidney tissue, with significant tubular swelling and glomerular damage in WT and Δ*sodA^C^* groups. Splenic tissue from infected groups showed increased immune cells in red pulp and macrophage proliferation. At 200 × magnification, in the Control group, the intestinal villi were neatly arranged, with intact glandular structures, and no significant pathological changes such as villus shedding or lamina propria edema were observed. The WT group exhibited the most severe lesions, characterized by sparse and fractured villi in the ileum, nearly absent crypts, a thinned intestinal wall, pronounced congestion, and inflammatory cell infiltration. In the colon, there was separation of the mucosal and lamina propria layers, severe damage to villus and crypt structures, and significant glandular injury accompanied by inflammatory cell infiltration. The Δ*sodA* group showed relatively intact intestinal villi, although they were sparse; the overall tissue morphology was more preserved compared to the WT group. The Δ*sodA^C^* group exhibited lesions more severe than the Δ*sodA* group, with partial destruction of villus structures, but less severe than the WT group.

### 3.9 Deletion of *sodA* gene affected the expression of NF-κB p65

**Figure 6:**
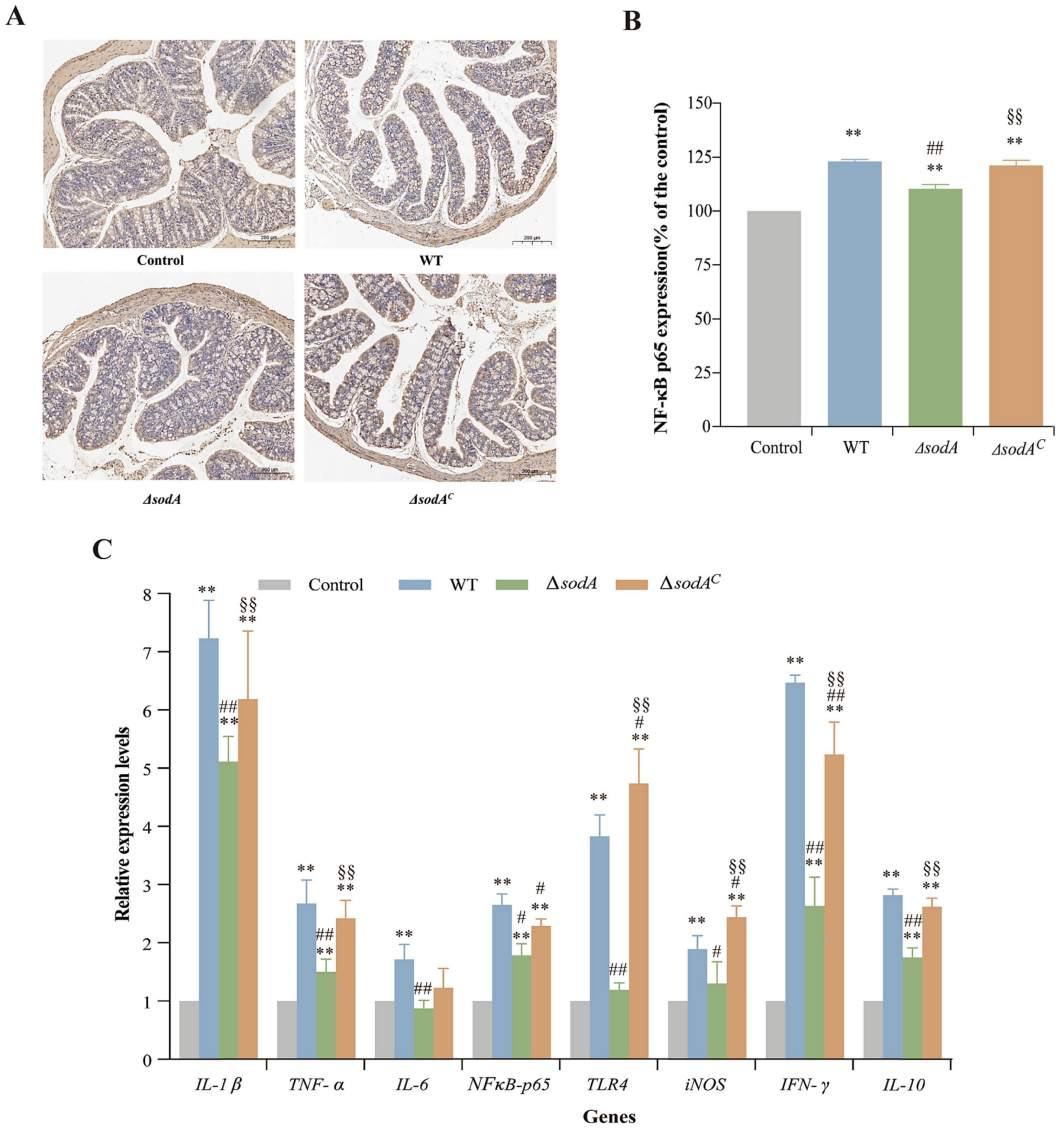
Immunohistochemical staining of NF-κB p65 in ileum tissues from mice **(A)** and relative expression of NF-κB p65 in ileum tissues from mice **(B)**. Relative mRNA transcription levels of inflammation-related genes in the colon tissue of mice by RT-qPCR **(C)**. * Indicates a comparison with the Control group (*: *p* < 0.05; **: *p* < 0.01); # indicates a comparison with the WT group (#: *p* < 0.05; ##: *p* < 0.01); § indicates a comparison with the Δ*sodA* group (§: *p* < 0.05; §§: *p* < 0.01). *n* = 3 biological replicates.

Immunohistochemical staining assessed NF-κB p65 expression in colon tissues (Figure 6A), with quantitative analysis using ImageJ (Figure 6B). Positive staining appeared as yellow or brown spots. WT, Δ*sodA*, and Δ*sodA^C^* groups showed significantly more brown spots than the control group, indicating enhanced inflammatory responses. Relative gray value analysis confirmed increased NF-κB p65 levels in infected groups (*p* < 0.01). The Δ*sodA* group exhibited lower NF-κB p65 expression compared to WT (*p* < 0.01). This suggests that the *sodA* gene deletion attenuates NF-κB pathway activation during *Y. enterocolitica* infection.

### 3.10 Deletion of *sodA* gene affected the expression of inflammation-related genes

The transcriptional levels of immune-related genes in mouse colons are shown in Figure 6C. The control group displayed low expression of inflammatory cytokines, whereas infection significantly upregulated genes such as IL-1*β*, TNF-*α*, NF-κB p65, INF-*γ*, and IL-10 (*p* < 0.01). The Δ*sodA* group showed reduced transcriptional levels of IL-1*β*, TNF-*α*, IL-6, TLR4, INF-*γ*, and IL-10 compared to WT (*p* < 0.01). No significant differences were observed in IL-6, iNOS, or TLR4 expression between Δ*sodA* and the control (*p* > 0.05). The Δ*sodA^C^* group displayed higher transcriptional levels than Δ*sodA*, with TLR4 and INF-*γ* being particularly elevated, reaching 4.74 and 5.24 times the control, respectively.

## 4 Discussion

*Y. enterocolitica* primarily infects the terminal ileum and proximal colon. Upon entering the host, the bacterium expresses the virulent pYV gene and secretes virulence factors, enabling colonization in the intestine and adhesion to intestinal epithelial cells (Bhunia, 2008). To successfully infect and invade the host, the pathogen must overcome the first line of host defense-phagocytosis by neutrophils and macrophages. Phagocytes generate reactive oxygen species (ROS) through respiratory bursts in response to pathogenic stimulation (Tang et al., 2012). The production of superoxide dismutase (SOD), encoded by the *sodA* gene, reduces superoxide anions, neutralizing the bactericidal effects of ROS. SOD functions as a virulence factor in certain pathogens, attenuating the oxidative burst effects of phagocytes (Tang et al., 2012). SODs have been implicated in survival and pathogenicity within phagocytes across multiple pathogens (Maurya and Namdeo, 2021). The study by Fu et al. (2014) confirmed that SODs, particularly copper-dependent SOD, helps pathogens (such as *Escherichia coli*, *Salmonella*, *Mycobacterium tuberculosis*, and *Streptococcus pneumoniae*) resist reactive oxygen species (ROS) toxicity induced by the host, thereby enhancing bacterial survival and pathogenicity. The study by Cavinato et al. (2020) demonstrates that SODB in *Pseudomonas aeruginosa* contributes to the production of lethal hydrogen peroxide early in infection, enhancing bacterial survival. However, in the later stages of infection, its role shifts to promoting bacterial survival, possibly through the activation of autophagy. Troxell et al. (2012) provided evidence that the SodA of *Borrelia burgdorferi* is a manganese-dependent superoxide dismutase, and that this enzyme helps the bacterium resist the accumulation of endogenous superoxide, enhancing its survival within the host. The creation of gene knockout mutants is essential for functional genomics studies in bacteria. Homologous recombination technology is commonly used to create gene knockout mutants, where exogenous DNA containing an antibiotic selection marker recombines with the homologous sequence of the target gene in the recipient cell’s chromosome (Tong et al., 2023). Gibson assembly is a seamless, efficient cloning method that allows the directional insertion of up to six DNA fragments into any vector site without requiring restriction sites. It boasts a high success rate, often yielding correct clones in a single attempt (Gibson et al., 2009). The *sodA* gene encodes an antioxidant enzyme (Mn-SOD), which helps the cell eliminate superoxide radicals and reduces oxidative stress-induced damage (Chiang and Schellhorn, 2012). However, the role of the *sodA* gene in *Y. enterocolitica* has not been systematically reported.

Our study demonstrated that *Y. enterocolitica* ATCC 23715 adheres to and invades epithelial cells while surviving and proliferating within macrophages. This ability to persist and replicate in RAW264.7 cells underpins the bacterium’s ability to evade host immune responses and antibiotic effects, potentially exacerbating infection. Furthermore, the deletion of the *sodA* gene weakened the ability of *Y. enterocolitica* to adhere to and invade Caco-2 cells (Figures 3A,B). These findings align with previous studies by McNally et al. (2006) and Zeitouni et al. (2016), which reported that *Y. enterocolitica* could penetrate and invade human intestinal epithelial cells (HEp-2 and Caco-2) and survive within macrophages. Similarly, Wang et al. (2018) found that *Salmonella enterica* Δ*sodA* mutants exhibited reduced adhesion and invasion abilities in epithelial cells compared to WT strains and were more sensitive to phagocytosis by RAW264.7 macrophages (Figure 3C). These results indicate that the pathogenicity of Δ*sodA* mutants is significantly diminished compared to WT strains. Poyart et al. (2001) showed that *Streptococcus agalactiae sodA* mutants were more susceptible to macrophage killing, corroborating our findings. These results suggest that Mn-dependent SODs mitigate host defenses against *Y. enterocolitica* by scavenging ROS in macrophages. The diminished pathogenicity observed in the Δ*sodA* mutants may be attributed to the reduced ability of the bacteria to neutralize reactive oxygen species (ROS) produced by host immune cells, which impairs their survival and virulence. By failing to effectively scavenge ROS, the mutants are more susceptible to oxidative stress and phagocytosis, leading to compromised colonization, invasion, and persistence within host tissues, as demonstrated by the reduced adhesion to and invasion of epithelial cells.

In a mouse infection model, *Y. enterocolitica* infection significantly reduced body weight, with WT strains causing more pronounced weight loss than Δ*sodA* mutants (Figures 4A,B). Colony counting revealed limited colonization in the liver and spleen, with higher bacterial loads in the ileum and colon. The Δ*sodA* mutant exhibited reduced colonization in the ileum and colon compared to WT and complemented strains, consistent with our cellular experiments (Figures 4C–E). These findings suggest that *sodA* is essential not only for effective adhesion and invasion of host cells but also for robust colonization of gastrointestinal tissues, particularly in the ileum and colon. The impaired adhesion, invasion, and colonization associated with *sodA* deletion highlight its critical role in the pathogenesis of *Y. enterocolitica*. H&E staining indicated that *Y. enterocolitica* infection disrupted intestinal villi structure and thinned the colonic lamina propria, with less severe intestinal damage observed in ΔsodA infections than in WT or complemented strains (Figure 5). Similarly, Poyart et al. (2001) showed that *S. agalactiae sodA* mutants exhibited lower bacterial loads in the blood and brain tissues of intravenously infected mice. Tang et al. (2012) observed that Δ*sodA* mutants of *Streptococcus suis* were more readily cleared in mouse infection models, showing a three-log reduction in bacterial loads in blood and tissues compared to WT strains. These findings suggest that *sodA* deletion reduces the colonization and pathogenicity of *Y. enterocolitica* in the host, particularly in the ileum and common, where the bacteria exert their pathogenic effects. These studies highlight the important role of SOD in mediating bacterial resistance to oxidative stress, which is essential for effective colonization and the development of infection.

By comparing the severity of lesions, it was observed that the ileum and colon exhibited more severe pathological changes. Given the thinner epithelium and higher shedding observed in the ileum, the colon tissue was selected for subsequent analyses. Immunohistochemical analysis revealed that *Y. enterocolitica* infection triggered a strong inflammatory response in the colon. Compared to WT and complemented strains, Δ*sodA* mutants showed reduced expression of NF-κB p65 protein in the colon (Figures 6A,B). NF-κB, a critical transcription factor in inflammation, regulates the stress response of inflammatory cells and the production of inflammatory mediators such as TNF-*α*, COX2, iNOS, IL-2, IL-6, IL-8, IL-12, chemokines, and adhesion molecules (Madboli and Seif, 2021). This suggests that *sodA* deletion may impair the inflammatory response by reducing NF-κB activation and the expression of pro-inflammatory genes, potentially altering the pathogenesis and severity of inflammation. The altered inflammatory response in Δ*sodA* mutants may have significant implications for the pathogenesis and severity of infection. A dampened inflammatory response can delay the recruitment of immune cells to the infection site, impair pathogen clearance, and potentially promote chronic or persistent infection. This is in line with studies that have shown that bacterial manipulation of NF-κB signaling can significantly influence disease outcomes. For example, *Salmonella enterica* has been shown to modulate NF-κB activation to survive within host macrophages and promote infection (Le Negrate et al., 2008). Similarly, *Pseudomonas aeruginosa* and other pathogens can exploit host inflammatory pathways to enhance their survival and virulence (Qin et al., 2022). Although attenuated NF-κB activation might theoretically assist Δ*sodA* in evading host clearance, this effect was negated by severe defects in adhesion and gut colonization. Thus, reduced fecal shedding of Δ*sodA* indicates inadequate colonization establishment, not enhanced colonic persistence.

RT-qPCR analysis further confirmed that *Y. enterocolitica* infection upregulated the expression of typical inflammatory genes such as IL-1*β*, TNF-*α*, NF-κB p65, IFN-*γ*, and IL-10. Compared to WT strains, the Δ*sodA* mutants showed lower transcriptional levels of inflammatory genes, including IL-1*β*, TNF-*α*, IL-6, TLR4, IFN-*γ*, and IL-10 (Figure 6C). Inflammation is a protective response involving various components of the host defense system, and pro-inflammatory cytokines play critical roles in regulating this process (Lodha et al., 2010). According to the study by Hsueh et al. (2003), intracellular TNF-*α* has a pro-inflammatory effect, increasing vascular permeability and exacerbating the inflammatory response. IL-1*β*, a pro-inflammatory cytokine, functions similarly to TNF-*α* by inducing the release of inflammatory mediators, activating inflammatory cells, and regulating the inflammatory factors of endothelial cells (Viscardi et al., 1997). IFN-*γ*, a key macrophage activator, is essential in combating pathogenic infections and is associated with inflammation and autoimmune diseases (Rottenberg et al., 2002). IL-10, an anti-inflammatory cytokine, modulates macrophage activity and suppresses the production of TNF-*β*, IFN-*γ*, and IL-2 (Latorre et al., 2013). Recent studies highlight the essential role of NF-κB in regulating inflammatory responses, thereby supporting the link between immunohistochemical analysis and the expression of inflammatory factors in this study. NF-κB signaling mediates the production of critical pro-inflammatory cytokines such as IL-1*β* and TNF-*α*, essential for immune cell recruitment and pathogen clearance (Lawrence, 2009). For instance, *Salmonella Typhimurium* uses effector proteins delivered through its type III secretion system to modulate NF-κB signaling, influencing cytokine production and inflammation during infection (Sun et al., 2016). The observed reduction in NF-κB p65 expression and pro-inflammatory cytokine levels in Δ*sodA* mutants in this study suggests impaired activation of NF-κB, which may account for the mutants’ diminished colonization and reduced inflammatory response. The upregulation of inflammatory genes in response to *Y. enterocolitica* infection and the reduced expression of these genes in Δ*sodA* mutants underscore the important role of SOD in modulating the host’s inflammatory response during infection.

The *sodA* gene of *Y. enterocolitica* likely contributes to the pathogen’s ability to regulate inflammatory cytokine expression, thereby influencing its infection capacity in the mouse intestine. Further studies are needed to elucidate the detailed regulatory mechanisms of immune-related cytokines, enzymes, and functional proteins during *Y. enterocolitica* infection. Our findings highlight the role of *sodA* in the pathogenesis of *Y. enterocolitica*, providing valuable insights into the pathological processes and laying a foundation for future therapeutic and preventive strategies.

## 5 Conclusion

This study demonstrates the role of the *sodA* gene in the pathogenicity of *Y. enterocolitica*. By encoding manganese-dependent superoxide dismutase (Mn-SOD), *sodA* helps the bacterium counteract host-derived reactive oxygen species (ROS), facilitating bacterial survival, colonization, and invasion. The Δ*sodA* mutants exhibited reduced colonization of the ileum and colon, lower inflammatory responses, and decreased virulence, underscoring the importance of *sodA* in host-pathogen interactions. These findings not only advance our understanding of *Y. enterocolitica* pathogenesis but also suggest that targeting ROS detoxification pathways, such as those mediated by *sodA*, could be a promising strategy for therapeutic intervention against bacterial infections.

## Data Availability

The raw data supporting the conclusions of this article will be made available by the authors, without undue reservation.

## Supplementary material

SUPPLEMENTARY FIGURE S1 Construction of *sodA* mutant and complemented strain.

SUPPLEMENTARY FIGURE S2 The impact of *sodA* deletion on acid tolerance, as measured by survival rates after 1 h exposure to pH 4.0.
