# Supplementary figures and images for: sodA modulates in vitro and in vivo virulence of Yersinia enterocolitica
Source: Front Microbiol. 2025 Sep 15;16:1643172. doi: 10.3389/fmicb.2025.1643172 (PMC12477148; doi:10.3389/fmicb.2025.1643172)

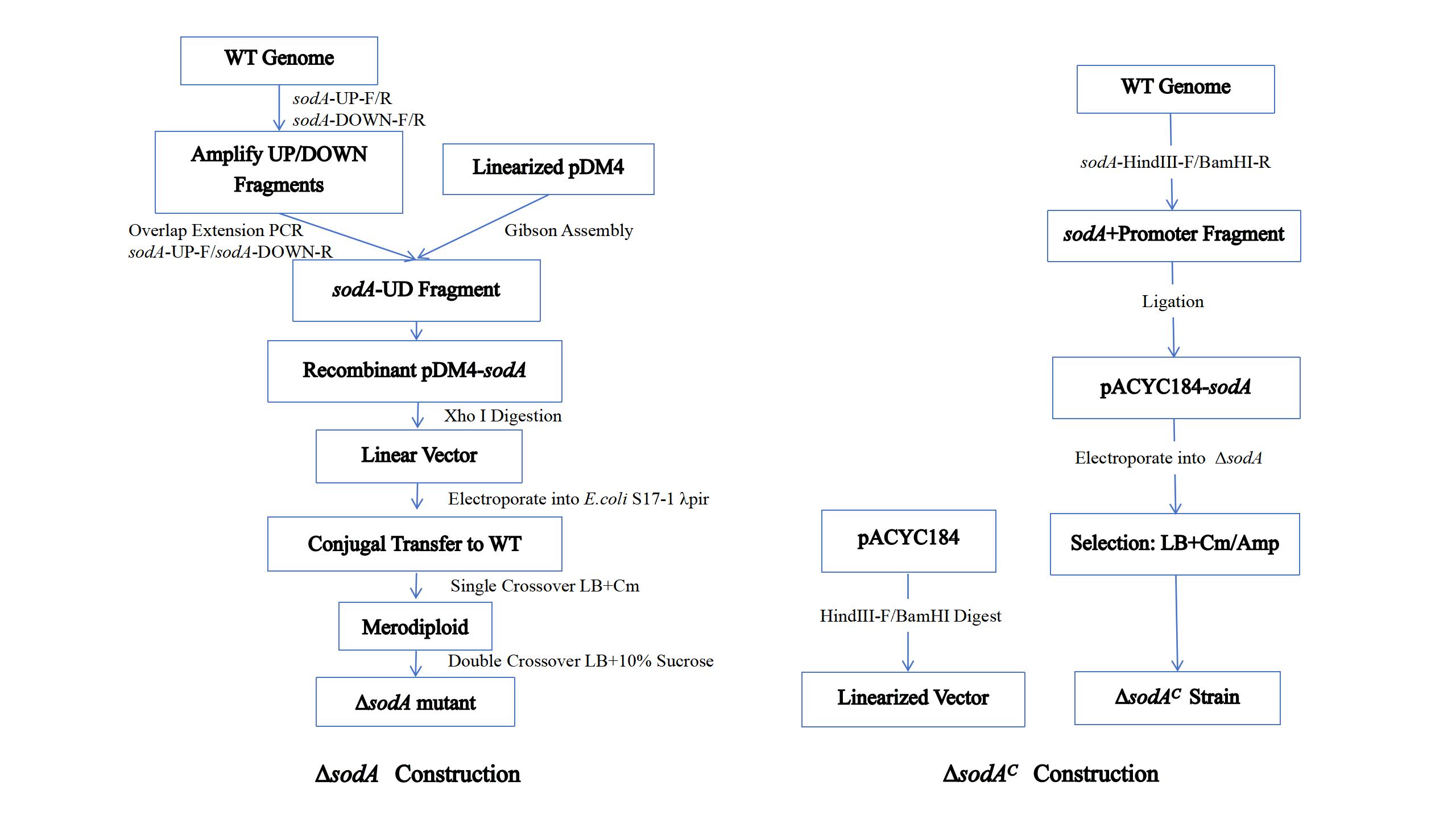

Supplement: SUPPLEMENTARY FIGURE S1 — Construction of sodA mutant and complemented strain. [file Image_1.JPEG]

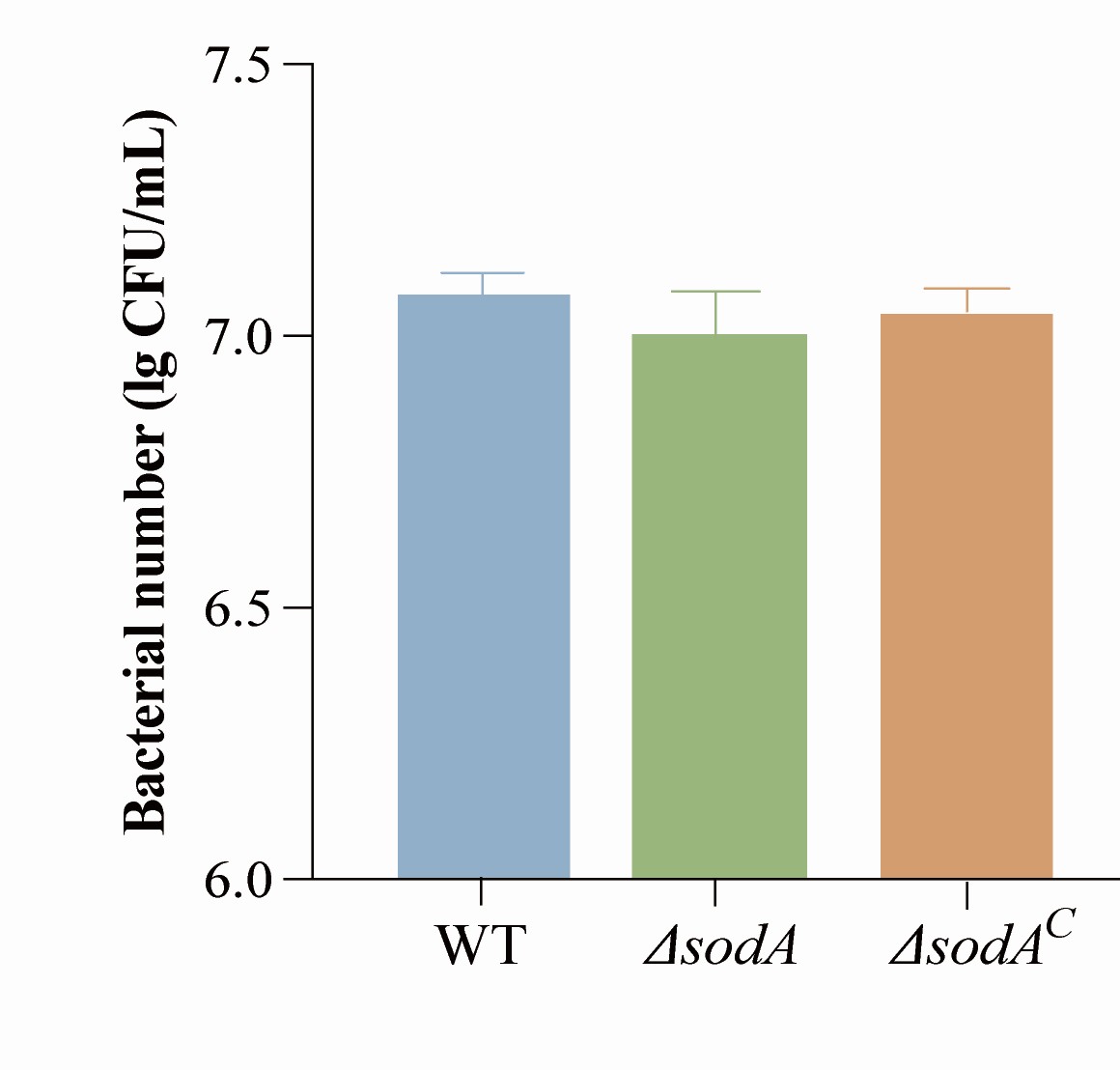

Supplement: SUPPLEMENTARY FIGURE S2 — The impact of sodA deletion on acid tolerance, as measured by survival rates after 1 h exposure to pH 4.0. [file Image_2.JPEG]
