# Supplementary material for: sodA modulates in vitro and in vivo virulence of Yersinia enterocolitica
Source: Front Microbiol. 2025 Sep 15;16:1643172. doi: 10.3389/fmicb.2025.1643172 (PMC12477148; doi:10.3389/fmicb.2025.1643172)
Supplement: Supplementary file 3 [file Table_1.DOCX]

**Table S1** The primers and gene descriptions used in this study

| **Primer** | **Sequence (5’-3’)** |
| --- | --- |
| *sodA*-UP-F | CACTAGTGACGCGTACTCGAGTGCCAGGCAACTTAACGTGCTACA |
| *sodA*-UP-R | TAGTCTGGGCGGGGCGTCATAAGCATAAGGCAGGGAT |
| *sodA*-DOWN-F | GCTTATGACGCCCCGCCCAGACTACATCAAAGCATTC |
| *sodA*-DOWN-R | GCTTATCGATACCGTCGACCCTCGAATTTCCGCCAGCAGAATCTGGGT |
| *sodA*-IN-F | GCGGCTGCTTCGTCCC |
| *sodA*-IN-R | TTACTCACTGCCATCCCTGC |
| *sodA*-HindIII-F | CCCAAGCTTACGCACGGTTATATTTCGCC |
| *sodA*-BamHI-R | CGCGGATCCCTCTGCCTTTCACTCAGACCT |

**Table S2.** Primers used for qRT-PCR

| **Gene** | **Sequence (5’-3’)** |
| --- | --- |
| GAPDH | F AGGTCGGTGTGAACGGATTTG  R TGTAGACCATGTAGTTGAGGTCA |
| IL-1β | F GCAACTGTTCCTGAACTCAACT  R ATCTTTTGGGGTCCGTCAACT |
| TNF-α | F CCCTCACACTCAGATCATCTTCT  R GCTACGACGTGGGCTACAG |
| IL-6 | F TAGTCCTTCCTACCCCAATTTCC  R TTGGTCCTTAGCCACTCCTTC |
| NF-κB p65 | F AGGCTTCTGGGCCTTATGTG  R TGCTTCTCTCGCCAGGAATAC |
| TLR4 | F ATGGCATGGCTTACACCACC  R GAGGCCAATTTTGTCTCCACA |
| iNOS | F TGGTCTTGCAAGCTGATGGTC  R ACCCACCTCCAGTAGCATGT |
| IFN- γ | F ATGAACGCTACACACTGCATC  R CCATCCTTTTGCCAGTTCCTC |
| IL-10 | F GGTTGCCAAGCCTTATCGGA  R ACCTGCTCCACTGCCTTGCT |
